# The Trem2 R47H Alzheimer’s risk variant impairs splicing and reduces Trem2 mRNA and protein in mice but not in humans

**DOI:** 10.1186/s13024-018-0280-6

**Published:** 2018-09-06

**Authors:** Xianyuan Xiang, Thomas M. Piers, Benedikt Wefers, Kaichuan Zhu, Anna Mallach, Bettina Brunner, Gernot Kleinberger, Wilbur Song, Marco Colonna, Jochen Herms, Wolfgang Wurst, Jennifer M. Pocock, Christian Haass

**Affiliations:** 1 Metabolic Biochemistry, Biomedical Center (BMC), Faculty of Medicine, Ludwig-Maximilians-Universität München, Munich, Germany; 2 Graduate School of Systemic Neuroscience, Ludwig- Maximilians- University Munich, Munich, Germany; 3 Department of Neuroinflammation, Cell Signalling Lab, University College London Institute of Neurology, WC1N 1PJ, London, UK; 4 German Center for Neurodegenerative Diseases (DZNE) Munich, Munich, Germany; 5 Munich Cluster for Systems Neurology (SyNergy), Munich, Germany; 6 Institute of Developmental Genetics, Helmholtz Zentrum München, German Research Center for Environmental Health, Neuherberg, Germany; 7 Department of Immunology and Pathology, Washington University in St. Louis, St. Louis, MO USA; 8 Center for Neuropathology and Prion Research, Ludwig-Maximilians-Universität München, Munich, Germany; 9 Technische Universität München-Weihenstephan, 85764 Neuherberg/Munich, Germany

**Keywords:** Alzheimer’s disease, Microglia, Neurodegeneration, TREM2, Pre-mRNA splicing, Human microglia

## Abstract

**Background:**

The R47H variant of the Triggering Receptor Expressed on Myeloid cells 2 (TREM2) significantly increases the risk for late onset Alzheimer’s disease. Mouse models accurately reproducing phenotypes observed in Alzheimer’ disease patients carrying the R47H coding variant are required to understand the TREM2 related dysfunctions responsible for the enhanced risk for late onset Alzheimer’s disease.

**Methods:**

A CRISPR/Cas9-assisted gene targeting strategy was used to generate Trem2 R47H knock-in mice. Trem2 mRNA and protein levels as well as Trem2 splicing patterns were assessed in these mice, in iPSC-derived human microglia-like cells, and in human brains from Alzheimer’s patients carrying the TREM2 R47H risk factor.

**Results:**

Two independent Trem2 R47H knock-in mouse models show reduced Trem2 mRNA and protein production. In both mouse models Trem2 haploinsufficiency was due to atypical splicing of mouse Trem2 R47H, which introduced a premature stop codon. Cellular splicing assays using minigene constructs demonstrate that the R47H variant induced abnormal splicing only occurs in mice but not in humans. TREM2 mRNA levels and splicing patterns were both normal in iPSC-derived human microglia-like cells and patient brains with the TREM2 R47H variant.

**Conclusions:**

The Trem2 R47H variant activates a cryptic splice site that generates miss-spliced transcripts leading to Trem2 haploinsufficiency only in mice but not in humans. Since Trem2 R47H related phenotypes are mouse specific and do not occur in humans, humanized TREM2 R47H knock-in mice should be generated to study the cellular consequences caused by the human TREM2 R47H coding variant. Currently described phenotypes of Trem2 R47H knock-in mice can therefore not be translated to humans.

**Electronic supplementary material:**

The online version of this article (10.1186/s13024-018-0280-6) contains supplementary material, which is available to authorized users.

## Background

Microgliosis has long been thought to play a central role in the initiation and progression of Alzheimer’s disease (AD) pathology. Indeed, genetic analyses recently revealed risk variants in a number of genes exclusively or at least preferentially expressed in microglia [1–5]. Among these, the gene encoding the triggering receptor expressed on myeloid cells 2 (TREM2) plays a pivotal role in regulating microglial activity [6–9]. As part of the disease associated signature of microglia (DAM; also called MGnD (microglia neurodegenerative disease)), TREM2 is one of the most upregulated genes when microglia encounter acute injuries within the brain or respond to neurodegenerative disorders such as AD and amyotrophic lateral sclerosis [6, 10]. Moreover, absence of functional TREM2 caused by a gene knockout or certain disease-associated sequence variants, which misfold TREM2 and retain the protein within the endoplasmic reticulum, lock microglia in a homeostatic state and prevent their activation in vivo [7, 11]. As a consequence cellular defense mechanisms such as chemotaxis, prominently visible by the lack of clustering around amyloid plaques, proliferation, phagocytosis of dead cells and amyloid fibrils are all reduced [8, 12, 13]. Furthermore, overexpression of human wild-type (wt) TREM2 in a Trem2 knockout mouse corrects loss-of-function phenotypes [14]. Thus TREM2 is believed to have protective functions. In line with that, TREM2 is upregulated early during disease development. In a study on patients with dominantly inherited AD (DIAN), soluble TREM2 was found to be increased 5 years before onset of clinical symptoms, which may also be interpreted as a protective response [15]. Consistent with this conclusion, lack of functional TREM2 affects amyloid plaque morphology and increases plaque associated neuritic dystrophies [13, 16]. Furthermore, in models of acute neuronal injury such as the cuprizone model, Trem2 activity facilitates clearance of cellular debris and recovery [11, 17, 18]. For tauopathies there are, however, opposing results indicating either protective or detrimental functions [19, 20]. In line with findings in mouse models for amyloid plaque pathology, this may be due to stage specific functions of Trem2.

Mouse models and cellular systems greatly helped to understand the consequences of loss-of-function mutations / haploinsufficiency of TREM2. However, the most important disease variant, namely R47H, which has been shown to increase the risk for late onset AD to a similar extent as the Apo lipoprotein E (ApoE) ε4 allele [3, 4], has been much less investigated. In cultured cells TREM2 R47H reduces ligand binding [8, 21–23]. Consistent with a pivotal role in ligand binding, structural analyses revealed that arginine 47 is required to stabilize a conformation, which is capable to interact with ligands such as ApoE and phosphatidylserine [24]. Furthermore maturation of the R47H variant within the secretory pathway may also be delayed [25]. In line with these findings, expression of human TREM2 R47H in Trem2 knockout mice failed to rescue their phenotypes [14]. These findings may therefore be indicative of a loss-of-function. In fact, very recently CRISPR/Cas9 generated mouse models expressing the R47H variant within the endogenous Trem2 mouse locus revealed a significant loss-of function [26, 27]. Trem2 R47H mice exhibited reduced Trem2 upregulation in microglia, reduced microgliosis, reduced clustering around amyloid plaques and an overall reduction of Trem2 protein [26]. Haploinsufficiency of Trem2 was confirmed by a significant reduction of Trem2 mRNA derived from the mutant allele [26]. Thus heterozygous Trem2 R47H mice appear to phenocopy a heterozygous knockout of Trem2.

We also independently generated Trem2 R47H knock-in mice using the CRISPR/Cas9 technology and reproduced haploinsufficiency of Trem2 in this model. Moreover, we could demonstrate that reduced mRNA stability due to a splicing error leads to a severe reduction of Trem2 mRNA. However, aberrant splicing was mouse specific and could not be observed in humans. Thus phenotypes associated with the R47H variant inserted into the endogenous mouse locus may not allow conclusions on the cellular mechanisms affected in humans.

## Methods

### Mice

Animal handling and animal experiments were performed in accordance to local animal laws and housed in standard cages in a specific pathogen-free facility on a 12-h light/dark cycle with ad libitum access to food and water.

Jax Trem2 R47H knock-in mice were purchased from Jackson laboratory. In-house Trem2 R47H knock-in mice were generated using CRISPR/Cas9 technology in C57BL/6 N background. Both strains were housed and bred in the same animal facility. To extract bone marrow, mice were first euthanized by CO_2_ followed by cervical dislocation.

### Generation of Trem2 R47H knock-in mice

Trem2 R47H knock-in mice (R47H ki mice) were generated by CRISPR/Cas9-assisted gene targeting in zygotes as described previously [28, 29]. Briefly, pronuclear stage zygotes were obtained by mating C57BL/6 N males with superovulated C57BL/6 N females (Charles River). Embryos were then microinjected into the male pronucleus with an injection mix containing 25 ng/μl Cas9 mRNA, 12.5 ng/μl Trem2-specific sgRNA, and 25 ng/μl single-stranded oligodeoxynucleotide (ssODN). Cas9 mRNA was prepared from XbaI-linerized pCAG-Cas9v2-162A by in vitro transcription using the mMESSAGE mMACHINE™ T7 ULTRA Transcription Kit (Thermo Fisher Scientific, #AM1345) and purified using the MEGAclear™ Transcription Clean-Up Kit (Thermo Fisher Scientific, #AM1908). Trem2-specific sgRNA (protospacer: GAAGCACTGGGGGAGACGCA) was prepared by IVT from pBS-T7-sgTrem2 using the MEGAshortscript™ T7 Transcription Kit (Thermo Fisher Scientific, #AM1354) and purified with the MEGAclear™ Transcription Clean-Up Kit. The 130 nt ssODN targeting molecule ssTrem2 R47H (5′- GGGCATGGCCGGCCAGTCCTTGAGGGTGTCATGTACTTATGACGCCTTGAAGCACTGGGG**TC**GACACAA**A**GCCTGGTGTCGGCAGCTGGGTGAGGAGGGCCCATGCCAGCGTGTGGTGAGCACACACGGT -3′), comprising the G > A substitution (underlined) and three additional silent mutations (bold), was synthetized by Metabion. After microinjection, zygotes were cultured in KSOM medium until they were transferred into pseudopregnant CD-1 foster animals.

### Off-target analysis of Trem2 R47H mice

To identify putative off-target sites of the Trem2-specific sgRNA, the online tool CRISPOR (http://crispor.tefor.net/) [30] was used. Predicted sites with a CFD score > 0.5 and an MIT score > 0.6 were chosen for off-target analysis. For analysis, genomic DNA of wildtype and heterozygous mutant Trem2 R47H mice was isolated and the loci were PCR amplified with primers flanking the putative cut sites. PCR amplicons were subsequently Sanger sequenced using PCR amplification primers and the traces compared to a reference sequence.

### Bone marrow derived macrophages culture

Bone marrow derived macrophages (BMDM) were prepared as previously described [31, 32]. Briefly, the bone marrow cells were flushed out using advanced RPMI 1640 (Life Technologies). Cells were differentiated using advanced RPMI 1640 supplemented with 2 mM L-Glutamine, 10% (*v*/v)) fetal calf serum (FCS), 100 U/ml penicillin, 100 μg/ml streptomycin and 50 ng/ml murine M-CSF (R&D System) for 7 days in non-cell culture treated dishes.

### Microglia isolation

Microglia were isolated as previously described with some modification [33]. Wild type and Trem2 R47H ki mice were perfused with cold phosphate buffered saline (PBS). Whole brain without the cerebellum was cut into small pieces and gently homogenized by mechanical dissociation in homogenization buffer (HBSS no calcium, no magnesium, with phenol red; 15 mM HEPES, 0.6% glucose). Homogenized cell suspensions were passed through a 100 μm cell strainer. Cells were pelleted at 300 g for 10 min and the supernatant discarded. To remove myelin, the cell pellets were re-suspended in 22% percoll (GE Healthcare) and centrifuged for 20 min at 900 g (acceleration 4, deceleration 0). Microglia were purified from the pellet using MACS CD11b magnetic beads according to manufacturer’s instructions. Briefly, 20 μl CD11b microbeads (Miltenyi) were incubated with cell suspension for 15 min. After incubation, 500 μl MACS buffer were added and cells were passed over a pre-rinsed Miltenyi LS column attached to a magnetic field. The column was washed three times with 500 μl MACS buffer, removed from the magnetic field and CD11b positive cells were washed out in 5 ml of MACS buffer.

### Cell lysis and immunoblotting

To detect membrane bound Trem2, membrane fractions were collected as previously described [32]. Briefly, cells were lysed in hypotonic buffer (100 mM Tris-HCl, pH 7.4, 1 mM EDTA, 1 mM EGTA, pH 7.4) freshly supplemented with a protease inhibitors cocktail (Sigma-Aldrich). Membrane fractions were pelleted by centrifugation for 45 min at 16,000 g at 4 °C. Membranes were lysed in STEN lysis buffer (150 mM NaCl, 50 mM Tris-HCl, pH 7.6, 2 mM EDTA, 1% Triton-X 100) on ice for 20 min. Equal amounts of protein were mixed with Laemmli sample buffer supplemented with β mercaptoethanol followed by by SDS-PAGE. Proteins were transferred onto polyvinylidene difluoride membranes (Amersham Hybond P 0.45 PVDF, GE Healthcare Life Science) and blocked in 10% I-BlockTM (Thermo Fisher Scientific) for 1 h. Monoclonal antibody 5F4 (dilution 1:100) [32] was used to detect Trem2.

### iPSC generation

Ethical permission for this study was obtained from the National Hospital for Neurology and Neurosurgery and the Institute of Neurology joint research ethics committee (study reference 09/H0716/64). R47H heterozygous fibroblasts were acquired with a material transfer agreement between University College London and University of California Irvine Alzheimer’s Disease Research Center (UCI ADRC; M Blurton-Jones). Fibroblast reprogramming was performed by episomal plasmid nucleofection (Lonza) as previously described [34], using plasmids obtained from Addgene (#27077, #27078 and #27080). Nucleofected cultures were transferred to Essential 8 medium (Life Technologies) after 7 days in vitro (DIV) and individual colonies were picked after 25–30 DIV. All iPSCs were maintained and routinely passaged in Essential 8 medium. Karyotype analysis was performed by The Doctors Laboratory (London, UK). Control iPSC lines used in this study are as follows: CTRL1 (kindly provided by Dr. Selina Wray, UCL Institute of Neurology); CTRL2 (SBAD03, Stembancc); CTRL3 (SFC840, Stembancc); CTRL4 (BIONi010-C, EBiSC).

### iPSC-derived microglia-like cells (iMG)

Using previously described protocols, iPSC-derived microglia-like cells (iMG) were generated [35, 36].

Day 0: iPSC lines were cultured to 60% confluency in E8 medium on vitronectin-coated 6-well plates. Cells were washed with PBS (w/o Ca2+/Mg2+), followed by trypLE digestion (1 ml/well; 4 min at 37 °C). The solution was added to 4 volumes of PBS (w/o Ca2+/Mg2+), and triturated to a single cell suspension. The suspension was centrifuged for 3 min at 300 g and the cell pellet resuspended in 1 ml EB differentiation medium (Adapted from [36]; EBdiff; Essential 8, 50 ng/ml BMP-4, 50 ng/ml VEGF, 20 ng/ml SCF, and 10 μM Y-27632). Cells were counted, and resuspended in EBDiff medium to a density of 10^5^ cells/ml. To generate embryoid bodies (EBs), 100 μl of the suspension was added to 96 well ultra-low attachment round bottom tissue culture plates (Corning), centrifuged at 115 g for 3 min, and transferred to a tissue culture incubator at 37 °C with 5% CO2.

Day 2: 50 μl of EBdiff medium was added to each well.

Day 3: Dense EBs were formed and collected with a P1000 Gilson pipette into a sterile 15 ml tube, and left to settle. The spent EBdiff medium was discarded and 10 ml of myeloid differentiation medium (Mdiff; X-VIVO 15 medium (Lonza), 1X Glutamax (Life Technologies), 100 U Penicillin/Streptomycin (Life Technologies), 50 μM β-mercaptoethanol (Life Technologies), 100 ng/ml MCSF (Peprotech), and 25 ng/ml IL-3 (Cell Guidance Systems)) added. Approximately 150 embryoid bodies (1.5 × 96 well plates) were transferred to a 175 cm2 flask containing a further 20 ml of Mdiff medium.

Day 9–11: Mdiff medium (30 ml) was added, to avoid acidosis, being careful not to disturb the EBs.

Day 26: Cells were collected from the medium for myeloid marker analysis.

Day 33: Once a week, 1/2 of the Mdiff medium/flask containing budded myeloid cells was collected through a 40 μm cell strainer (Falcon, Corning), replacing the collected medium with fresh Mdiff. The myeloid cell suspension was centrifuged for 3 min at 300 g and the cell pellet resuspended in 1 ml microglial differentiation medium (Adapted from [35]; MGdiff; DMEM/F12 HEPES no phenol red, 2% ITS-G (Life Technologies), 1% N2 supplement (Life Technologies), 200 μM monothioglycerol (Sigma), 1X Glutamax, 1X NEAA (Life Technologies), 5 μg/ml Insulin (Sigma), 100 ng/ml IL34 (Peprotech), 25 ng/ml MCSF and 5 ng/ml TGFβ1 (Peprotech), filtered through a 0.22 μm syringe filter. Cells were counted and plated in MGdiff, and medium replaced every 3–4 days.

Day 46: MGdiff medium was replaced with microglial maturation medium (MGmat: MGdiff + 100 ng/ml CD200 (Generon), and 100 ng/ml CX3CL1 (Peprotech)) for 4 days to generate iMG.

### Isolation of human blood derived-monocytes

Monocytes were obtained from blood through centrifugation with Histopaque (Sigma) to isolate peripheral blood mononuclear cells followed by separation and purification with CD14-conjugated magnetic beads (Miltenyi). Peripheral blood monocytes (PBM) were matured into monocyte-derived macrophages (hMacs) in X-VIVO 15 medium with 1% Glutamax, 100 U Penicillin/Streptomycin, and 100 ng/ml MCSF for 7 days.

### Microglia signature gene array

A custom gene array based on published microglial expression data [35, 37–39] was used to confirm a microglial signature in our iMG cultures (TaqMan Array Plate 32 plus Candidate Endogenous Control Genes; Thermo Fisher Scientific). Complementary DNA was generated from iMG, iPSC-derived microglial-like cells [36], and human monocyte-derived macrophages (hMacs) RNA samples using the High-Capacity RNA-cDNA kit (Life Technologies), according to the manufacturer’s instructions. Human primary microglia cDNA was also analyzed as a control sample (ScienCell). Quantitative PCR were conducted on the Mx3000p qPCR system with MxPro qPCR software (Stratagene) using TaqMan Gene Expression Mastermix (Thermo Fisher Scientific). Heat maps were generated with the gplots [40] and d3heatmap [41] packages in R.

Microglial gene signature primer details:

**Table:** 

Gene name	Primer ID
18 s rRNA	Hs99999901_s1
GAPDH	Hs99999905_m1
HPRT	Hs99999909_m1
GUSB	Hs99999908_m1
APOE	Hs00171168_m1
C1QA	Hs00706358_s1
C1QB	Hs00608019_m1
ITGAM	Hs00167304_m1
CSF1R	Hs00911250_m1
CX3CR1	Hs01922583_s1
GAS6	Hs01090305_m1
GPR34	Hs00271105_s1
AIF1	Hs00610419_g1
MERTK	Hs01031979_m1
OLFML3	Hs01113293_g1
PROS1	Hs00165590_m1
SALL1	Hs01548765_m1
SLCO2B1	Hs01030343_m1
TGFBR1	Hs00610320_m1
TMEM119	Hs01938722_u1
TREM2	Hs00219132_m1
BIN1	Hs00184913_m1
CD33	Hs01076282_g1
SPI1	Hs02786711_m1
HEXB	Hs01077594_m1
ITM2B	Hs00222753_m1
C3	Hs00163811_m1
A2M	Hs00929971_m1
C1QC	Hs00757779_m1
RGS1	Hs01023772_m1
FTL	Hs00830226_gH
P2RY12	Hs01881698_s1

### Immunocytochemistry

Cells were fixed in 4% PFA/sucrose in PBS for 20 min at room temperature (RT), quenched with 50 mM NH_4_Cl in PBS for 10 min at RT, and permebalized with 0.2% Triton X-100 in PBS for 5 min at RT. Blocking was performed with 5% normal goat serum (NGS) in PBS for 30 min. Primary antibodies were diluted in 5% NGS/PBS and incubated at RT for 2 h, followed by PBS washes and incubation with corresponding secondary antibodies for 1 h at RT in the dark, with gentle rocking. Nuclei were counterstained during mounting using Vectorshield with DAPI (4′, 6-diamidino-2-phenylindol; Vector Labs). Fluorescence microscopy was performed on a Zeiss Axioskop 2 microscope and Axiovison software (Zeiss, v4.8). Confocal microscopy was performed on a Zeiss LSM 710 confocal microscope using Zen software (Zeiss, Version 2012), and all images were processed with Image J1.51 K (https://imagej.nih.gov/ij/). The following antibodies were used: mouse anti-CD68 (1:100, DAKO), rabbit-anti-P2YR12 (1:200, Atlas Antibodies), mouse-anti-β-Actin (1:500, Sigma), mouse-anti-EZR (1:250, Atlas Antibodies), goat anti-rabbit Alexafluor488 (1:500, Life Technologies), goat-anti-mouse Alexafluor568 (1:500, Life Technologies).

### Cellular splicing assay

Human and mouse Trem2 genomic fragments encoding exon 1, intron 1, exon 2, and a FLAG tag were synthesized by Integrated DNA Technologies. The following primer sets were used for vector cloning: EcoRV-Hs TREM2-Fw (GGATATCCGGGCAGCGCCTGACATGCCTG) and NotI-Hs TREM2-Rv (ATGCGGCCGCTTAGGATTACAAGGATGACGACGATAAG); HindIII-Mm Trem2 Fw (CCCAAGCTTGGGGCGCCTACCCTAGTCC) and XohvI-Mm Trem2-Rv (CCGCTCGAGCGGCTACTTGTCGTCATCG). The amplified fragments were digested by EcoRV/NotI or HindIII/XohI and the digested fragments were inserted into pcDNA3.1 (+) (Invitrogen). The mutations were introduced in the human and mouse minigene using the Quikchange™ site-directed mutagenesis kit (Agilent). The following mutants were generated:

1. R47H is the G > A variant alone encoding arginine;
2. R47H^TCA^ corresponds to sequence variants expressed by the in-house generated Trem2 R47H ki mice (GA > TC, G > A, G > A; also see Fig. 4b);
3. R47H^AA^ represents the Trem2 R47H ki mice generated by Jackson Laboratory (G > A, G > A, C > A; also see Fig. 4b);
4. TC are the silent mutations in in-house generated Trem2 R47H ki mice (GA > TC)
5. AA are the silent mutations in Jax R47H ki mice (G > A, C > A).
6. R47H^T^ represents Trem2 R47H ki mice generated by Cheng Hathaway et al. [26]

For the cellular splicing assay 8 × 10^5^ HEK293 cells were seeded in 6-well plates with Dulbecco’s Modified Eagle Medium (Life Technologies) supplemented with GlutaMAX™, 10% (*v*/v) FCS, 100 U/ml penicillin, 100 μg/ml streptomycin and cultured overnight. Cells were transfected with 3 μg of plasmids to express human or mouse Trem2 minigenes using 6 μl lipofectamine 2000 according the manufacturer’s instructions (Thermo Fisher Scientific). Transfected cells were cultured in normal medium for 48 h and collected for RNA extraction using RNeasy Mini Kit (Qiagen) according the manufacturer’s instructions. The RNA was used for Reverse transcription polymerase chain reaction (RT-PCR).

### Reverse transcription polymerase chain reaction

1 μg of total RNA was transcribed into cDNA using SuperScript IV reverse transcriptase and oligo dT (Thermo Fisher Scientific). 2 μl of cDNA was used as template and amplified by polymerase chain reaction (PCR) with GoTaq DNA polymerase (Promega) according the manufacturer’s instructions. The reaction condition and primer sequences are listed in Table 1. The PCR products were loaded into 2% agarose gel with GelRed™ (Biotium) for DNA visualization.

**Table 1:** Primers and PCR conditions used for detecting splice pattern (Forward: Fwd; Reverse: Rev)

Name	Sequence	Reaction condition
MmTrem2 Fwd	GCTCAATCCAGGAGCACAGT	95 °C 1 min; (95 °C 30s, 65 °C 15 s, 72 °C 1 min) *35 cycle; 72 °C 10 min
MmTrem2 Rev	TCTGACACTGGTAGAGGCCC	
HsTREM2 Fwd	GCCTGACATGCCTGATCCTC	95 °C 1 min; (95 °C 30s, 65 °C 15 s, 72 °C 1 min) *35 cycle; 72 °C 10 min
HsTREM2 Rev	AGGACCTTCCTGAGGGTGTC	

### Quantitative real-time polymerase chain reaction

RNeasy Mini Kit (Qiagen) was used for total RNA isolation according the manufacturer’s instructions. 1 μg of total RNA was transcribed into cDNA using SuperScript IV reverse transcriptase and oligo dT (Thermo Fisher Scientific). RNA levels of human and mouse Trem2 and Tyrobp were analyzed by Taqman® real-time PCR using the 7500 Fast real-time PCR system (Applied Biosystems). For endogenous controls Gusb (Mm01197698_m1, Thermo Fisher Scientific) and Hsp90ab1 (Mm00833431_g1, Thermo Fisher Scientific) or GUSB (Hs00939627_m1, Thermo Fisher Scientific) and HSP90AB1 (Hs03043878_g1, Thermo Fisher Scientific) were used. Probes that target mouse Trem2 and Tyrobp are Mm04209424_g1, Mm04209423_g1, Mm01273682_g1, Mm00449152_m1 (Thermo Fisher Scientific). Probes that target human TREM2 and TYROBP are Hs01010721_m1, Hs01003899_m1, Hs00182426_m1 (Thermo Fisher Scientific). For allele specific mRNA expression custom made primers and probes were designed. The primer pair for mouse Trem2 is: CCTTGAGGGTGTCATGTACTTAT and TCCCATTCCGCTTCTTCAG. The probes for the mouse wild-type allele and R47H allele are /5HEX/CCTT+G + C + GT + CT + CC/3IABkFQ/ (+, lock nucleic acid) and /56-FAM/CTT + T + G + T + GT + C + GA + C/3IABkFQ/, respectively (Integrated DNA Technologies). The primer pair for human TREM2 is ACAAGTTGTGCGTGCTGA and ATGACTCCATGAAGCACTGG. The probes for the human wild-type allele and the R47H allele are /5HEX/CTT + G + C + GCCT+CC/3IABkFQ/ and /56-FAM/TT + G + T + GC + CT + CC/3IABkFQ/, respectively (Integrated DNA Technologies). The probes and primers were mixed in 1:2 ratios for quantitative PCR reaction.

cDNAs were diluted 1:5 with H_2_O and 9 μl of diluted cDNA together with 1 μl of primer probe mix, 10 μl of Taqman® master mix (Thermo Fisher Scientific) were used in one 20 μl reaction.

## Results

### Reduced mRNA and protein level in Trem2 R47H knock-in mice

**Fig. 1:**
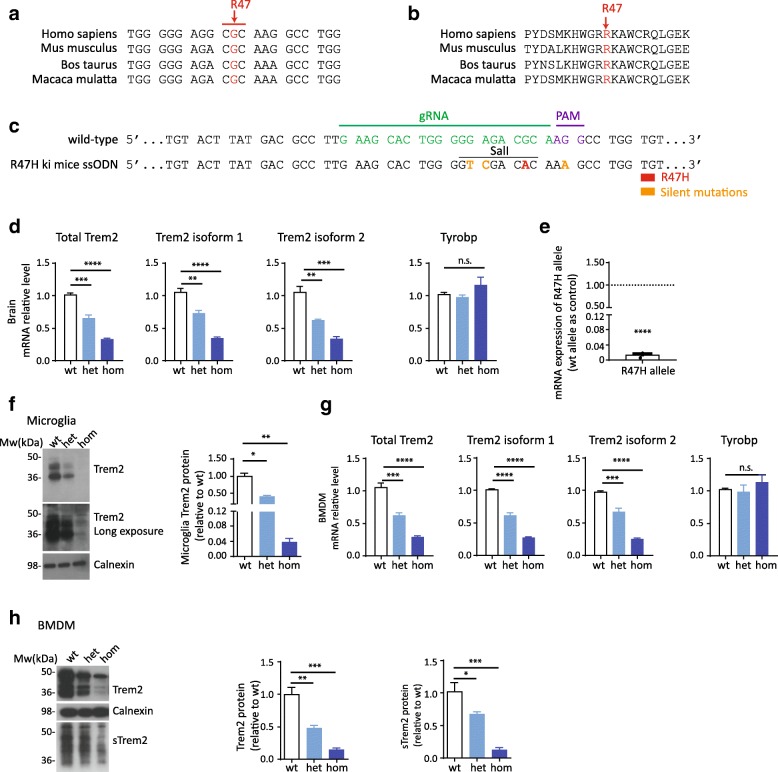
Trem2 mRNA and protein are reduced in a novel Trem2 R47H knock-in mouse model. **a** and **b** Evolutionary conservation of TREM2 at the DNA (**a**) and protein (**b**) level. **c** Strategy to generate Trem2 R47H knock-in (R47H ki) mice indicating the protospacer region (green), protospacer adjacent region (PAM, purple), and the introduced nucleotide changes (orange or red). The restriction site for SalI is underlined. **d** Trem2 and Tyrobp mRNA levels in brains from R47H ki mice. TaqMan probes for the exon 4/5 boundary were used to detect total Trem2 mRNA. TaqMan probes for the Trem2 exon 3/4 boundary were used for isoform discrimination. (*N* = 3, +/-SEM, one way ANOVA, Bonferroni-corrected pair-wise post hoc tests, total Trem2 WT vs. Het *p* = 0.0002, WT vs. Hom *p* < 0.0001; Trem2 isoform 1 WT vs. Het *p* = 0.0029, WT vs. Hom *p* < 0.0001; Trem2 isoform 2 WT vs. Het *p* = 0.0031, WT vs. Hom *p* = 0.0002. n.s. Non-significant). **e** Allele specific Trem2 mRNA expression in heterozygous R47H ki mice. Customized probes were against Trem2 R47H and its neighbor region (see also Methods). (N = 3, +/-SEM, unpaired t test, *p* < 0.0001). **f** Trem2 protein expression in microglia isolated from Trem2 wt or R47H ki mice. (N = 3,+/-SEM, one way ANOVA, *p* < 0.0001, Bonferroni-corrected pair-wise post hoc tests, WT vs. Het *p* = 0.0005, WT vs. Hom *p* < 0.0001). **g** Trem2 and Tyrobp mRNA levels in bone marrow derived macrophages (BMDM) isolated from Trem2 wt and R47H ki mice. (N = 3, +/-SEM, one way ANOVA, Bonferroni-corrected pair-wise post hoc tests, Trem2 WT vs. Het *p* = 0.0002, WT vs. Hom *p* < 0.0001; Trem2 isoform 1 WT vs. Het *p* < 0.0001, WT vs. Hom *p* < 0.0001; Trem2 isoform 2 WT vs. Het *p* = 0.0008, WT vs. Hom *p* < 0.0001. n.s. Non-significant.) **h** Expression levels of membrane bound and soluble Trem2 (sTrem2) protein in BMDM isolated from Trem2 wt or R47H ki mice. (N = 3, +/-SEM, one way ANOVA, Trem2 *p* = 0.0003, Bonferroni-corrected pair-wise post hoc tests, WT vs. Het *p* = 0.0026, WT vs. Hom *p* = 0.0002, sTrem2 *p* = 0.0007, WT vs. Het *p* = 0.0469, WT vs. Hom *p* = 0.0005)

The rare TREM2 variant rs75932628-T encodes a histidine instead of arginine at position 47 (R47H) and increases the risk for AD around three-fold [3, 4]. The DNA and amino acid sequence around arginine at position 47 is highly conserved across different mammalian species (Fig. 1a and b). To study the impact of this variant on TREM2 function in microglia in vivo we generated Trem2 R47H knock-in mice (R47H ki mice) by introducing a G > A mutation using the CRISPR/Cas9 technology (Fig. 1c). Two silent mutations were additionally introduced (GA > TC) to create a SalI restriction site for genotyping (Fig. 1c). In addition a silent G > A mutation was generated to block the protospacer-adjacent motif (PAM) to allow higher gene editing efficiency (Fig. 1c). The predicted potential off-target sites were analyzed. An off-target event with a Δ10-Indel mutation was identified at an intragenic region on chromosome 11 (Additional file 1: Figure S1, putative off-target #2). The founder mouse was back-crossed to C57BL/6 N, and off-spring with positive Trem2 R47H ki but negative off-target #2 (i.e ID-7-1; ID-7-2; ID-7-4) were used for establishing the mouse line. Expression of total Trem2 mRNA as well as both Trem2 mRNA transcripts (NM_031254.3 and NM_001272078.1), encoding either membrane bound Trem2 or a truncated soluble version were validated in brain. Interestingly, total Trem2 mRNA including both Trem2 transcripts was significantly reduced in a gene dose-dependent manner, whereas mRNA expression of Tyrobp (NM_011662), the adaptor protein of Trem2 [42], remained unchanged (Fig. 1d). Using allele specific qPCR we confirmed that expression of the R47H allele was selectively reduced compared to the wt allele in heterozygous R47H ki mice (Fig. 1e and Additional file 2: Table S1).

To confirm decreased Trem2 expression on protein level, we purified microglia from wild-type (wt), heterozygous (het), and homozygous (hom) R47H ki mice and performed western blotting of membrane fractions. Membrane-bound Trem2 showed a gene dose-dependent reduction (Fig. 1f).

Reduced Trem2 R47H mRNA and protein expression was further confirmed in bone marrow derived macrophages (BMDM). Consistent with our findings in brain and microglia, mRNA of both Trem2 transcripts decreased in a gene dose dependent manner whereas mRNA of Tyrobp remained unchanged (Fig. 1g). Furthermore, immature and mature Trem2 as well as soluble Trem2 (sTrem2) were also reduced in a gene dose dependent manner (Fig. 1h).

**Fig. 2:**
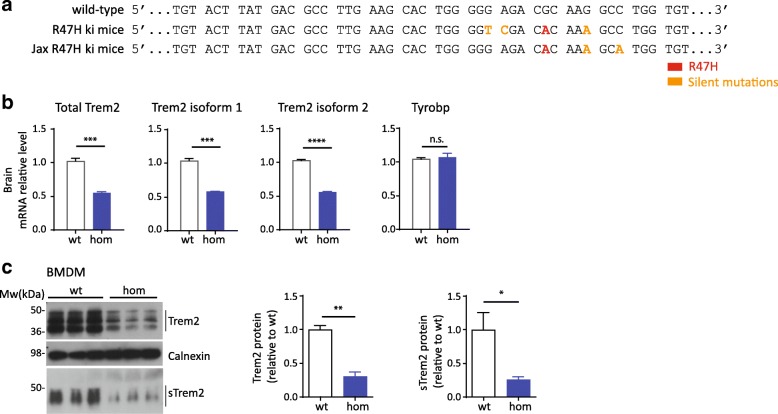
Trem2 haploinsufficiency in an independent R47H knock-in mouse model provided by Jackson laboratories. **a** DNA sequence comparison of in-house made Trem2 R47H ki mice (R47H ki mice) and Jax Trem2 R47H ki mice (Jax R47H ki mice). **b** Trem2 and Tyrobp mRNA levels in brains of wt or Jax R47H ki mice. (*N* = 3, +/-SEM, unpaired t test, Total Trem2 *p* = 0.0005; Trem2 isoform1 *p* = 0.0002; Trem2 isoform2 *p* = 0.0001. n.s. Non-significant.). **c** Expression levels of membrane bound and soluble Trem2 (sTrem2) protein in bone marrow derived macrophages (BMDM) isolated from Trem2 wt or Jax R47H ki mice (*N* = 3, +/-SEM, unpaired t test, Trem2 *p* = 0.0016, sTrem2 *p* = 0.0433)

To exclude that reduced Trem2 mRNA and protein expression is artificially caused by the introduction of the three silent mutations, we analyzed Trem2 R47H knock-in mice generated by Jackson laboratory (Jax R47H ki mice). In addition to the target variant R47H, these mice harbor two silent mutations (Fig. 2a). One of the silent mutations is a G > A exchange to block the PAM sequence exactly like in the mice generated within our laboratory. The second silent mutation C > A is only present in the Jax R47H ki mice (Fig. 2a). We investigated Trem2 mRNA and protein levels using homozygous Jax R47H ki mice and their wt counterparts. Again, mRNA from total Trem2 as well as from both transcripts of Trem2 decreased in brains of homozygous ki mice whereas mRNA expression of Tyrobp remained unchanged (Fig. 2b). Western blotting of membrane fractions from BMDM from the Jax R47H ki mice also confirmed a significant reduction of membrane bound and soluble Trem2 (Fig. 2c). Thus reduction of mRNA and protein levels in Trem2 R47H ki mouse was confirmed in two independent mouse models.

### Aberrant splicing of exon1/2 in Trem2 R47H knock-in mice

**Fig. 3:**
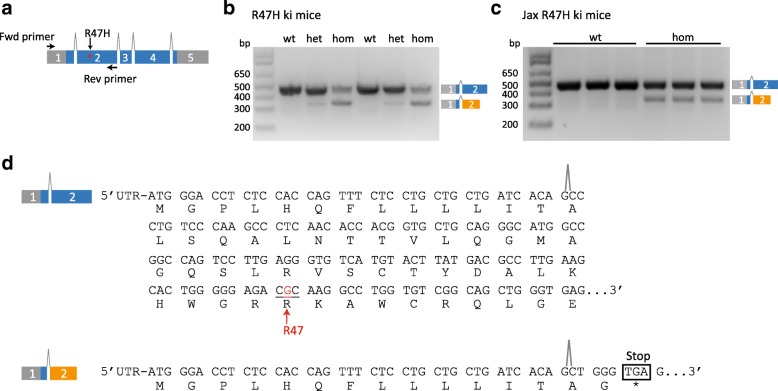
Aberrant splicing of exon1/2 in two independent Trem2 R47H knock-in mice. **a** Schematic representation of exon/intron boundaries of Trem2 and the strategy used to investigate exon 1/2 splicing. **b** RT-PCR mediated amplification of splicing products generated by R47H ki mice. **c** RT-PCR mediated amplification of splicing products generated by Jax R47H ki mice. **d** DNA and amino acid sequence of the two splice products identified. Fwd: Forward; Rev: Reverse

Next we searched for the cellular mechanism responsible for the substantial reduction of the Trem2 R47H mRNA in the two mouse models. Since nonfunctional mRNAs are rapidly removed by mRNA surveillance systems [43], we studied splicing of the first intron which separates exon 2 containing the R47H variant from exon 1 harboring the translation initiation site and upstream untranslated sequences. Using RT-PCR with primer pairs against the 5′ end of exon 1 and the 3′ end of exon 2, we compared the splicing patterns of Trem2 in R47H ki and wt mice (Fig. 3a). A single splicing product of the expected length (465 base pairs) was obtained from wt Trem2 mRNA (Fig. 3b). Surprisingly, an additional smaller splicing product (346 base pairs) was observed in Trem2 R47H heterozygous mice, which was even more abundant in Trem2 R47H homozygous mice (Fig. 3b) while at the same time the larger splicing product was reduced in a gene dose dependent manner (Fig. 3b). This suggests aberrant splicing of Trem2 pre-mRNA derived from the mutant allele. To independently confirm aberrant splicing, we investigated the Jax R47H ki mice described in Fig. 2. Again, we found an additional smaller RT-PCR product in mutant but not wt mice (Fig. 3c). Furthermore, the larger splicing product was reduced in homozygous ki mice (Fig. 3c). Thus aberrant splicing of R47H pre-mRNA occurs in two independent mouse models. DNA sequencing of the splicing products revealed correct splicing of exon 1 and exon 2 in wt mice and to a lesser extent also in the ki mice (Fig. 3d). However, DNA sequencing of the additional shorter RT-PCR product of both mutant mice revealed that 119 base pairs were deleted at the 5′ end of exon 2 (Fig. 3d and Additional file 3: Figure S2). The deletion leads to a frame shift and a premature stop codon in exon 2 (Fig. 3d), which may lead to nonsense mediated mRNA decay and thus explain the consistent reduction of the mutant mRNA in both mouse models.

### The R47H variant does not affect splicing and mRNA levels in humans

**Fig. 4:**
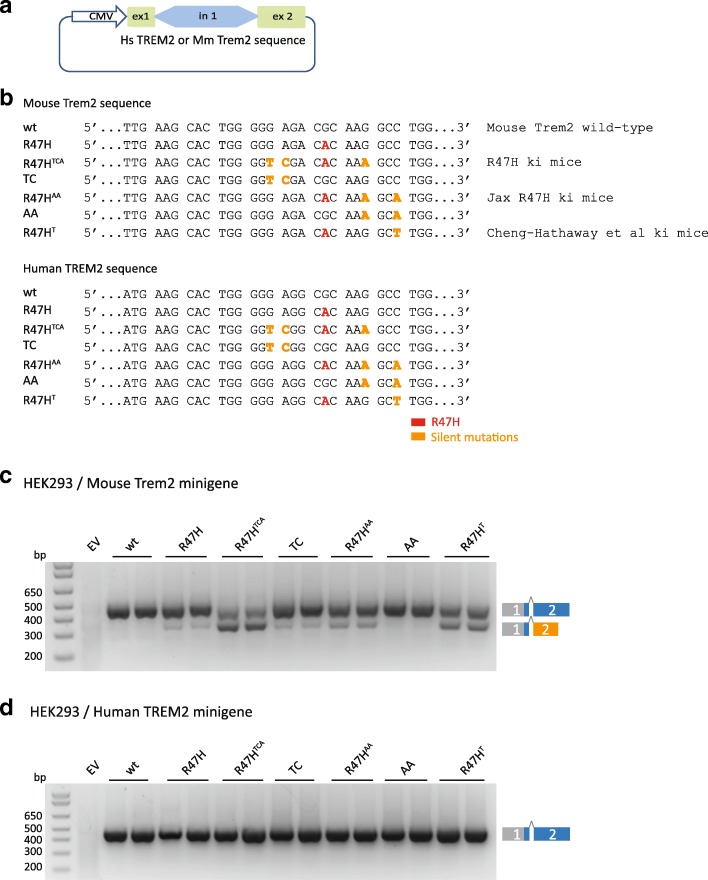
Aberrant splicing of Trem2 variants containing the R47H mutation with and without additional mutations used to create three different R47H ki mice. **a** The minigene construct used to investigate exon1/2 splicing of the Trem2 variants shown in (**b**). **b** Sequence alignment of Trem2 variants investigated for aberrant splicing. **c** Exon 1/2 splicing of mouse Trem2 variants described in (**b**). **d** Exon 1/2 splicing of human TREM2 variants described in (**b**). Note that only mouse transcripts undergo aberrant splicing. EV: empty vector

To directly compare the splicing pattern of mouse and human TREM2 R47H, we expressed mouse or human TREM2 minigenes containing exon 1, intron 1, and exon 2 in human embryonic kidney 293 cells (HEK 293) (Fig. 4a). To separately investigate the disease causing TREM2 R47H variant and the silent mutations, we introduced the corresponding sequence variants either alone or together (Fig. 4b). Upon expression of the minigene encoding the mouse Trem2 sequence, we observed miss-splicing induced by the R47H variant alone (R47H) (Fig. 4c). Similar aberrant splicing was observed when the two silent mutations of the Jax R47H ki mice were expressed in addition to the R47H variant (R47H^AA^), whereas introduction of the two silent mutations alone (AA) did not affect splicing (Fig. 4c). Moreover, when the three silent mutations used to generate our in-house mouse model were combined with the R47H (R47H^TCA^), a striking increase of aberrant splicing was observed. When we only introduced the unique TC mutations used to generate our mouse model (TC) we also observed impaired splicing (Fig. 4c), suggesting that R47H and TC together display synergistic effects on aberrant splicing. This demonstrates that the R47H variant by itself is sufficient to induce aberrant splicing, but enhanced miss-splicing upon the addition of silent mutations implies that this genomic region is very sensitive for splicing errors induced by minor changes within the pre-mRNA sequence. Finally, we also investigated if the mutations introduced into the mouse genome by Cheng-hathaway et al. [26] affect pre-mRNA splicing of Trem2. Very similar to our R47H ki mice and those generated by the Jackson Laboratories, the mutations introduced by Cheng-hathaway and colleagues (R47H^T^) caused aberrant splicing (Fig. 4c), which is consistent with the haploinsuffciency also observed in this model [26].

To prove if this is also true for human TREM2, we investigated the same TREM2 variants in human minigene constructs (Fig. 4a, b and d). Surprisingly, our results demonstrate that neither the R47H variant alone or in combination with the silent mutations used to generate the ki mouse models affected correct exon1/2 splicing (Fig. 4d). This suggests that only the mouse gene locus is vulnerable for aberrant splicing upon introduction of these sequence variants and implies that the R47H mutation does not affect splicing and mRNA levels in humans.

**Fig. 5:**
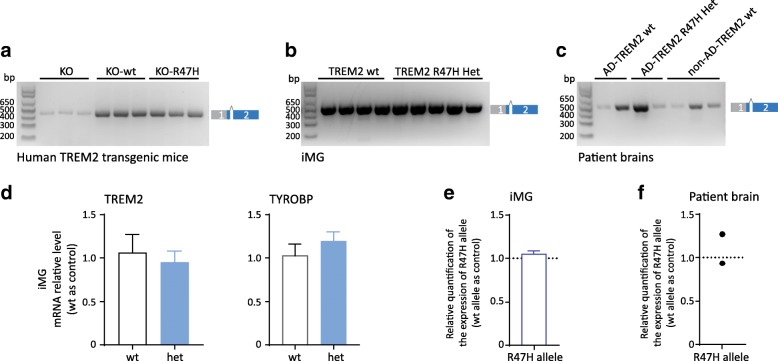
Normal exon 1/2 splicing of human TREM2 pre-mRNA encoding the R47H variant. **a** Normal splicing of human TREM2 upon ectopic expression of the human wt or R47H mutant TREM2 locus in Trem2^−/−^ mice. **b** Normal exon 1/2 splicing of Trem2 in human induced pluripotent stem cell (iPSC)-derived microglia-like cells (iMG) with the wt TREM2 allele or heterozygous for the TREM2 R47H variant. **c** No aberrant splicing of the R47H variant in an AD case carrying one R47H mutant allele. **d** No reduction of total TREM2 mRNA in iMG with one R47H allele. (*N* = 4, +/-SEM, unpaired t test, non-significant.) **e** Allele specific qPCR demonstrates that the expression of the R47H allele is comparable to the wt allele in iMG. (*N* = 7, +/-SEM) **f** Allele specific qPCR demonstrates that the expression of the R47H allele is comparable to wt allele in human brains derived from R47H carriers. (*N* = 2). Customized probes were against Trem2 R47H and its neighbor region (see also Methods)

To provide direct evidence for this prediction, we investigated exon 1/2 splicing in humanized TREM2 mice generated by ectopic expression of the human wt or R47H mutant TREM2 locus in Trem2^−/−^ mice [14]. Using the same RT-PCR strategy as described above (Fig. 3a), we could only detect the correctly spliced exon 1 and 2 but no aberrantly spliced additional products (Fig. 5a).

Similarly, in human induced pluripotent stem cell (iPSC)-derived microglia-like cells (iMG) with the wt TREM2 allele or heterozygous for the TREM2 R47H variant (Additional file 4: Figure S3) we also detected only the correctly spliced exon 1 and 2 (Fig. 5b). Furthermore, no aberrant splicing was detected in AD cases carrying one R47H mutant allele (Fig. 5c). Direct sequencing demonstrated correct exon1/2 splicing in human iMG and in AD cases. Lack of aberrant splicing of human TREM2 R47H is consistent with no reduction of total TREM2 mRNA in iMG with one R47H allele (Fig. 5d). In addition, using allele specific qPCR we confirmed that the expression of the R47H allele is comparable to wt in iMG (Fig. 5e and Additional file 5: Table S2), and in human brain (Fig. 5f and Additional file 5: Table S2). Taken together, aberrant splicing of R47H mutant pre-mRNA is not observed in humans and consequently no haploinsufficiency of TREM2 could be detected.

## Discussion

Most functional studies of TREM2 have so far been performed with either total loss-of-function models or by studying the TREM2 T66M mutation [7, 8, 11–13, 19, 20, 44, 45], which is associated with an FTD-like syndrome. Homozygous mice expressing the Trem2 T66M variant phenocopy a number of functional deficits also observed upon total loss of the Trem2 encoding gene [11]. These include delayed resolution of inflammation upon lipopolysaccharide stimulation, reduced phagocytic activity, reduced microglial activation during physiological ageing and neurological insults, reduced cerebral blood flow, reduced cerebral brain glucose metabolism, impaired chemotaxis and clustering of microglia around amyloid plaques [11]. However, much less is known about the functional impact of the TREM2 R47H variant, which is associated with a high risk for AD similar to that caused by the ApoE ε4 allele [3, 4, 46]. In vitro studies suggested reduced binding of Aβ oligomers, ApoE, and phosphatidylserine due to structural alterations in TREM2 [8, 21–24]. Furthermore maturation of the R47H variant within the secretory pathway may also be delayed [25]. Expression of human TREM2 R47H in Trem2 knockout mice failed to rescue the knockout phenotypes again supporting the notion that TREM2 is protective and that TREM2 variants associated with neurodegenerative diseases may cause a loss-of-function [14]. Trem2 R47H knock-in mice expressing this variant under physiological conditions resulted in phenotypes that were compatible with a loss-of function [26]. Cheng-Hathaway et al. reported that Trem2 R47H heterozygous mice showed reduced Trem2 expression in microglia close to amyloid plaques, reduced microglial proliferation, reduced dense core plaques and increased neuritic dystrophy [26]. These phenotypes were associated with reduced Trem2 mRNA expression [26]. Similarly, Sudom et al. reported reduced Trem2 protein expression in brain as well as reduced sTrem2 in plasma from R47H knock-in mice [24]. All together, this suggests that the AD-associated Trem2 R47H variant is also associated with a loss-of-function. However, while the T66M mutation causes a loss-of-function due to retention of the misfolded mutant protein within the endoplasmatic reticulum [11, 25], the R47H mutation appears to cause haploinsufficiency due to reduced mRNA levels [26]. We generated a similar mouse model using the CRISPR/Cas9 technology. Consistent with published findings, our mouse model also showed reduced Trem2 mRNA and protein levels. Furthermore, similar findings were made using an independent mouse generated by Jackson Laboratories. We therefore searched for a joined cellular mechanism, which may be involved in the significant reduction of Trem2 mRNA and protein in both mouse models. Surprisingly, we found that the introduction of the R47H variant by itself but even more so the introduction of additional silent mutations caused aberrant splicing. Direct sequencing of the aberrant splicing product revealed that it lacks 119 base pairs. Close inspection of the sequence of the alternative splice product revealed that a cryptic splice acceptor site within the exon 2 was activated. This leads to the elimination of parts of exon 2 resulting in a splicing product containing a premature stop codon. It is well known that such aberrant mRNAs are rapidly degraded by nonsense-mediated mRNA decay [47]. Note that the TaqMan probes used for detecting Trem2 mRNA were either bound to exon 3/4 or exon 4/5 boundary (Fig. 1d) thus they detect both the full length functional Trem2 mRNA and the aberrantly spliced shorter variant. Therefore, the apparent degree of reduction for Trem2 mRNA and protein is not equal (Fig. 1d and f). However, we cannot fully exclude the possibility that the R47H variant may also affect mouse Trem2 protein stability.

The mRNA reductions caused by aberrant splicing, which were consistent in two independent mouse models, led us to investigate if aberrant exon 2 splicing in humans also leads to TREM2 haploinsufficiency. We found that TREM2 mRNA levels and splicing patterns were both normal in iPSC-derived human microglia-like cells and in patient brains with the TREM2 R47H variant. Furthermore, cellular splicing assays using minigene constructs demonstrate that the R47H variant induced abnormal splicing only occurs in mice but not in humans. Thus, both the R47H variant as well as additionally introduced silent variants cause a mouse-specific reduction of Trem2 mRNA. Therefore, these findings cannot be translated to humans calling for novel humanized R47H mouse models.

## Conclusions

The AD-associated Trem2 R47H variant in combination with silent mutations introduced by the CRISPR/Cas9 technology causes mouse specific aberrant splicing of exon 2, which leads to an alternative Trem2 mRNA containing a premature stop codon. The observed significant reduction of Trem2 mRNA and protein in R47H ki mice is most likely the consequence of nonsense-mediated mRNA decay of the aberrant transcript and is not observed in human systems. Thus, functional data derived from Trem2 R47H knock-in mice cannot be translated to humans.

## Additional files

Additional file 1: **Figure S1.** Off-target analysis of in-house made Trem2 R47H knock-in mice. **a** Sanger-sequencing chromatograms of the Trem2 on-target site and the six putative off target sites of animal Trem2 R47H ki ID-7. Mixed peaks in the Trem2 locus show the correct R47H substitution (CGC > CAC) and the three silent mutations for genotyping purposes. Mixed peaks in traces of site #2 reveal a Δ10-Indel mutation at the putative cut site, indicating a true off target event. Underlined: Protospacer; arrow head: putative cut site; green letters: PAM site on shown strand; red letters: PAM site on complementary strand; yellow: Δ10-Indel mutation. **b** Sanger sequencing results of Trem2 R47H positive off-springs of male ID-7, which was crossed with a C57BL/6 N female. The Δ10-Indel allele was inherited to animals ID-7-3 und ID-7-12 that were excluded from any further breedings and experiments. (PDF 201 kb)

Additional file 2: **Table S1.** Allele specific quantitative PCR for Trem2 R47H knock-in mice (PDF 92 kb)

Additional file 3: **Figure S2.** Sequence of the aberrantly spliced murine Trem2 mRNA. Trem2 gene sequence. Exon in black; intron in green; // indicates splicing sites. (PDF 93 kb)

Additional file 4: **Figure S3.** iMG differentiation and validation. **a** Schematic of the in vitro differentiation of iPSC-derived microglia-like cells (iMG). (i): Human iPSCs are grown in feeder free conditions with no spontaneous differentiation. Scale bar: 250 μm. (ii): Embryoid bodies are formed in the presence of 3 factors SCF, BMP4, and VEGF; Scale bar: 750 μm. (iii): Myeloid cells are generated after culturing with IL3 and MCSF growth factors for 3–4 weeks, then stained for classic myeloid/macrophage markers CD68. Scale bar: 50 μm. (iv): Further differentiation to microglia-like cells that positive for microglial markers P2RY12. Scale bar: 20 μm. (v): Addition of the two final factors (CD200 and CX3CL1) matures the iMG. Scale bar: 20 μm. **b** Heat map showing mRNA expression of a microglial gene signature in iMG, human monocyte-derived macrophages (hMac), human primary microglia (hMG), and iPSC samples. Clear clustering is observed between iMG and hMG. (PDF 159 kb)

Additional file 5: **Table S2.** Allele specific quantitative PCR for iMG and patient brains (PDF 104 kb)

## Abbreviations

AD: Alzheimer’s disease
Aβ: Amyloid β-peptide
BMDM: Bone marrow derived macrophages
HEK293: Human embryonic kidney 293
Hom: Homozygous
iMG: iPSC derived microglia-like cells
iPSC: Induced pluripotent stem cell
ki: Knock-in
PAM: Protospacer-adjacent motif
qPCR: Quantitative polymerase chain reaction
RT-PCR: Reverse transcriptase polymerase chain reaction
sTREM2: Soluble TREM2
TREM2: Triggering receptor expressed on myeloid cell 2
WT: Wild-type; Het: heterozygous
