# Supplementary figures and images for: The Trem2 R47H Alzheimer’s risk variant impairs splicing and reduces Trem2 mRNA and protein in mice but not in humans
Source: Mol Neurodegener. 2018 Sep 6;13:49. doi: 10.1186/s13024-018-0280-6 (PMC6126019; doi:10.1186/s13024-018-0280-6)

A

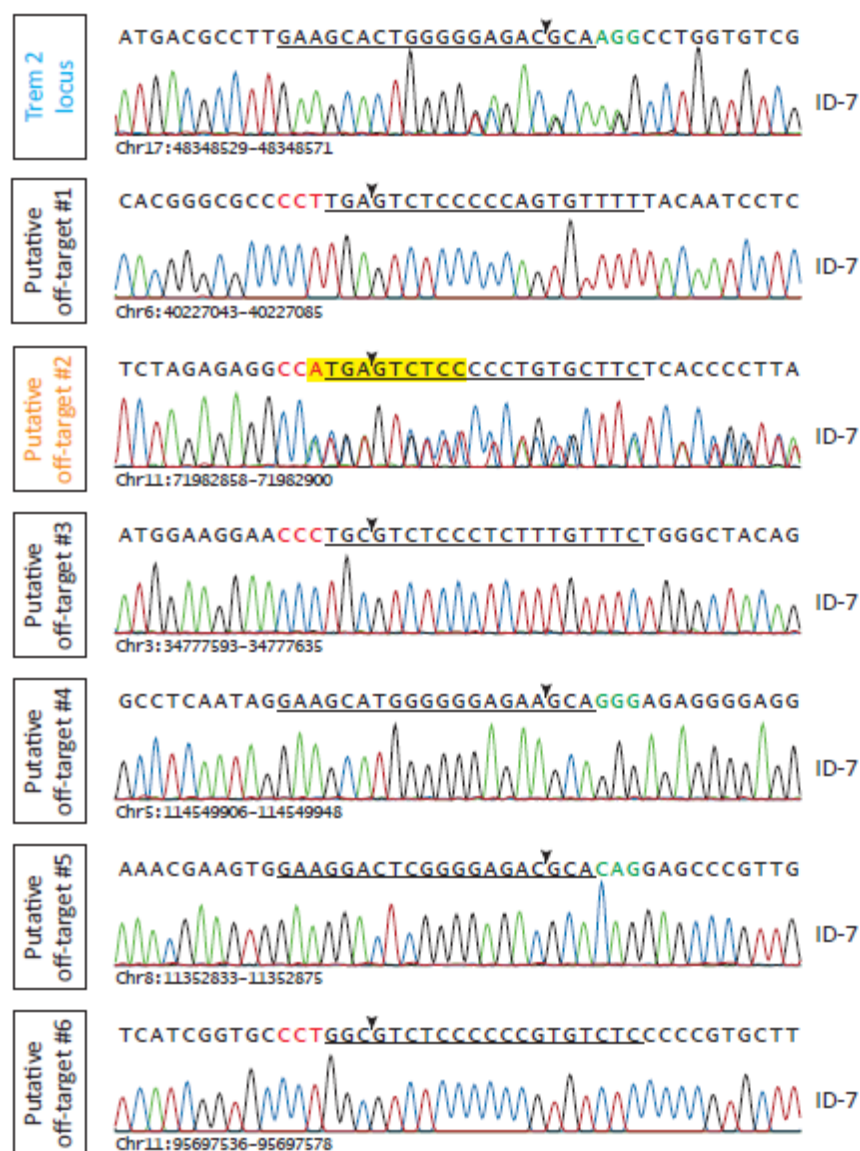

B

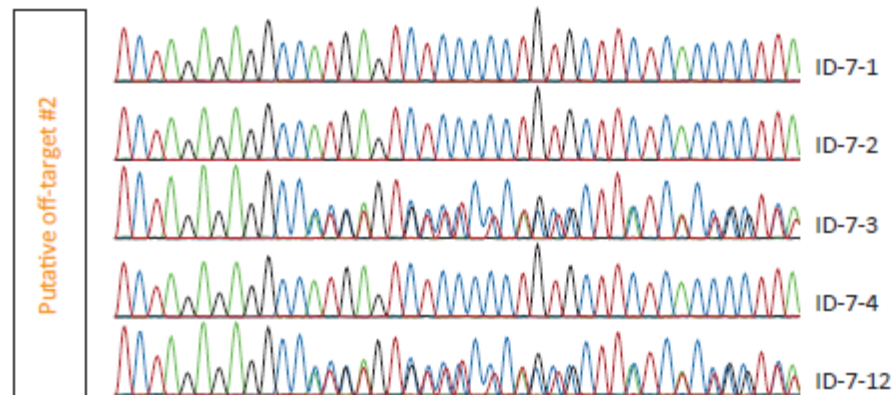

Supplement: Supplementary file 1 — Figure S1. Off-target analysis of in-house made Trem2 R47H knock-in mice. a Sanger-sequencing chromatograms of the Trem2 on-target site and the six putative off target sites of animal Trem2 R47H ki ID-7. Mixed peaks in the Trem2 locus show the correct R47H substitution (CGC > CAC) and the three silent mutations for genotyping purposes. Mixed peaks in traces of site #2 reveal a Δ10-Indel mutation at the putative cut site, indicating a true off target event. Underlined: Protospacer; arrow head: putative cut site; green letters: PAM site on shown strand; red letters: PAM site on complementary strand; yellow: Δ10-Indel mutation. b Sanger sequencing results of Trem2 R47H positive off-springs of male ID-7, which was crossed with a C57BL/6 N female. The Δ10-Indel allele was inherited to animals ID-7-3 und ID-7-12 that were excluded from any further breedings and experiments. (PDF 201 kb) [file 13024_2018_280_MOESM1_ESM.pdf]

a

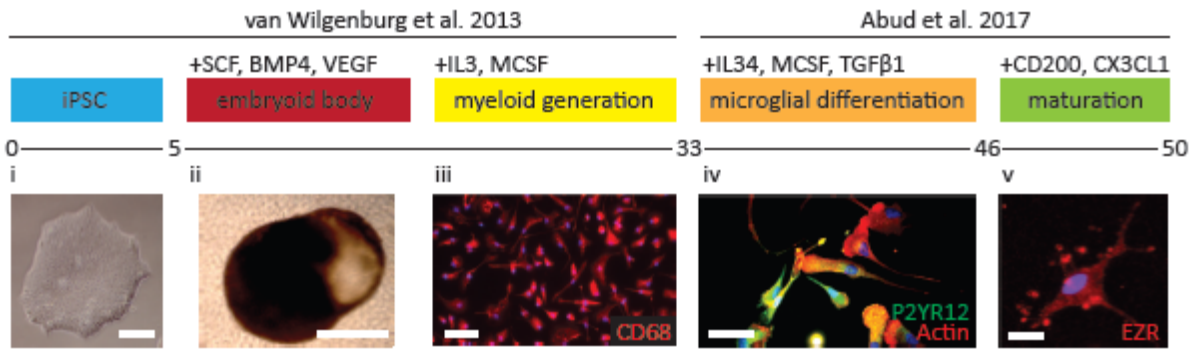

b

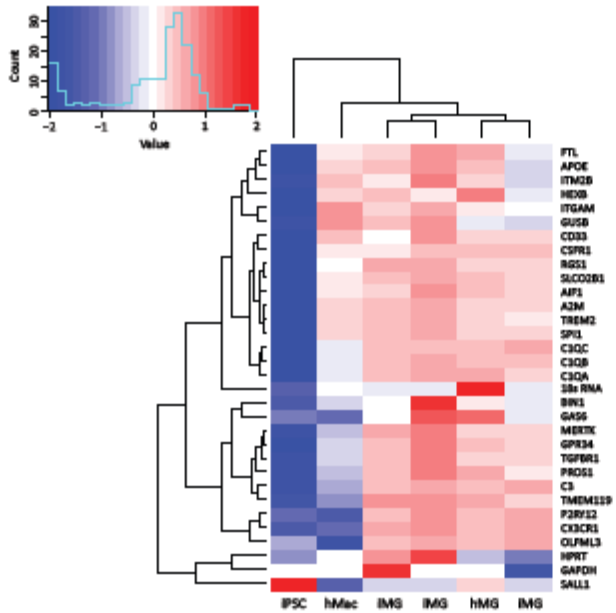

Supplement: Supplementary file 4 — Figure S3. iMG differentiation and validation. a Schematic of the in vitro differentiation of iPSC-derived microglia-like cells (iMG). (i): Human iPSCs are grown in feeder free conditions with no spontaneous differentiation. Scale bar: 250 μm. (ii): Embryoid bodies are formed in the presence of 3 factors SCF, BMP4, and VEGF; Scale bar: 750 μm. (iii): Myeloid cells are generated after culturing with IL3 and MCSF growth factors for 3–4 weeks, then stained for classic myeloid/macrophage markers CD68. Scale bar: 50 μm. (iv): Further differentiation to microglia-like cells that positive for microglial markers P2RY12. Scale bar: 20 μm. (v): Addition of the two final factors (CD200 and CX3CL1) matures the iMG. Scale bar: 20 μm. b Heat map showing mRNA expression of a microglial gene signature in iMG, human monocyte-derived macrophages (hMac), human primary microglia (hMG), and iPSC samples. Clear clustering is observed between iMG and hMG. (PDF 159 kb) [file 13024_2018_280_MOESM4_ESM.pdf]
