# Supplementary material for: The Trem2 R47H Alzheimer’s risk variant impairs splicing and reduces Trem2 mRNA and protein in mice but not in humans
Source: Mol Neurodegener. 2018 Sep 6;13:49. doi: 10.1186/s13024-018-0280-6 (PMC6126019; doi:10.1186/s13024-018-0280-6)
Supplement: Supplementary file 2 — Table S1. Allele specific quantitative PCR for Trem2 R47H knock-in mice (PDF 92 kb) [file 13024_2018_280_MOESM2_ESM.pdf]

Table S1. Allele specific quantitative PCR for Trem2 R47H knock-in mice

Ct mean			
	WT allele	R47H allele	RQ
	16,00	Undetermined	-
	15,98	Undetermined	-
WT	16,12	Undetermined	-
	Undetermined	19,92	-
	Undetermined	19,64	-
R47H hom	Undetermined	19,24	-
	17,22	24,68	0,0057
	17,43	23,20	0,0183
R47H het	17,02	23,00	0,0159
