# Supplementary material for: The Trem2 R47H Alzheimer’s risk variant impairs splicing and reduces Trem2 mRNA and protein in mice but not in humans
Source: Mol Neurodegener. 2018 Sep 6;13:49. doi: 10.1186/s13024-018-0280-6 (PMC6126019; doi:10.1186/s13024-018-0280-6)
Supplement: Supplementary file 3 — Figure S2. Sequence of the aberrantly spliced murine Trem2 mRNA. Trem2 gene sequence. Exon in black; intron in green; // indicates splicing sites. (PDF 93 kb) [file 13024_2018_280_MOESM3_ESM.pdf]

a

Normal splicing of Trem2 exon 1 and 2

5′ UTR-ATGGGACCTCTCCACCAGTTTCTCCTGCTGCTGATCACAG//**GT**GGGACCACAGGAGGGAC
CTA...TGTCTCCTCTGC**AG**//CCCTGTCCCAAGCCCTCAACACCACGGTGCTGCAGGGCATGGCCGG
CCAGTCCTTGAGGGTGTCATGTACTTATGACGCCTTGAAGCACTGGGGGAGACGCAAGGCCTGGTGTC
GGCAGCTGGGTGAGGAGGGCCCATGCCAGCGTGTGGTGAGCACACACGGTGTGTGGCTGCTGGCCTTC
CTGA...

Aberrant splicing of Trem2 exon 1 and 2

5′ UTR-ATGGGACCTCTCCACCAGTTTCTCCTGCTGCTGATCACAG//**GT**GGGACCACAGGAGGGAC
CTA...TGTCTCCTCTGCAGCCCTGTCCCAAGCCCTCAACACCACGGTGCTGCAGGGCATGGCCGGCC
AGTCCTTGAGGGTGTCATGTACTTATGACGCCTTGAAGCACTGGGGGAGACGCAAGGCCTGGTGTCGG
C**AG//**CTGGGTGAGGAGGGCCCATGCCAGCGTGTGGTGAGCACACACGGTGTGTGGCTGCTGGCCTTC
CTGA... Stop
