# Supplementary material for: The Trem2 R47H Alzheimer’s risk variant impairs splicing and reduces Trem2 mRNA and protein in mice but not in humans
Source: Mol Neurodegener. 2018 Sep 6;13:49. doi: 10.1186/s13024-018-0280-6 (PMC6126019; doi:10.1186/s13024-018-0280-6)
Supplement: Supplementary file 5 — Table S2. Allele specific quantitative PCR for iMG and patient brains (PDF 104 kb) [file 13024_2018_280_MOESM5_ESM.pdf]

Table S2. Allele specific quantitative PCR for human TREM2 materials

	Ct Mean		
	WT allele	R47H allele	RQ
iMG TREM2 wt	19,922	Undetermined	-
	19,122	Undetermined	-
	26,041	Undetermined	-
	19,616	Undetermined	-
BAC mice TREM2 wt	19,005	Undetermined	-
	19,199	Undetermined	-
BAC mice TREM2 R47H Hom	Undetermined	17,296	-
	Undetermined	17,628	-
iMG TREM2 R47H Het	20,790	20,737	1,038
	20,074	20,061	1,009
	20,674	20,589	1,061
	19,804	19,905	0,932
	21,68	21,63	1,034
	20,89	20,65	1,177
	21,68	21,63	1,034
TREM2 R47H Het patient brain	26,99	27,09	0,934
	31,20	30,86	1,270
